
JOURNAL INFORMATION
==============================
Journal ID: ijcoms

IJQHC Communications

EISSN: 2634-5293

ARTICLE INFORMATION
==============================
PMCID: PMC8344599
DOI: 10.1093/ijcoms/lyab011
Article ID: lyab011
Article version: 1
Subjects: Commentary, AcademicSubjects/MED00862

COVID-19 and Changes in Healthcare in North America

Bates David W MD, MSc Division of General Internal Medicine and Primary Care, Department of Medicine, Brigham and Women’s Hospital, Boston, MA, USA
Harvard Medical School, Boston, MA, USA
Department of Health Policy and Management, Harvard T. H. Chan School of Public Health, Boston, MA

Correspondence: dbates@bwh.harvard.edu
Electronic publication date: 2021 Jul 13
Electronic Location ID: lyab011
Received 2021 Jun 3; Revised 2021 Jul 2; Accepted 2021 Jul 12; 2021 Jul 8
Copyright: © The Author(s) 2021. Published by Oxford University Press in association with International Society for Quality in Health Care.
Copyright year: 2021
License: This is an Open Access article distributed under the terms of the Creative Commons Attribution License (http://creativecommons.org/licenses/by/4.0/), which permits unrestricted reuse, distribution, and reproduction in any medium, provided the original work is properly cited.
License URL: https://creativecommons.org/licenses/by/4.0/

Keywords: Pandemics, quality, safety
edited-state: accepted-manuscript
article-lifecycle: PAP

==============================
### COVID-19 and Changes in Healthcare in North America

David W. Bates, MD, MSc[1,2,3,]

[1] Division of General Internal Medicine and Primary Care, Department of Medicine, Brigham and Women's Hospital, Boston, MA, USA
[2] Harvard Medical School, Boston, MA, USA
[3] Department of Health Policy and Management, Harvard T. H. Chan School of Public Health, Boston, MA

**Correspondence**: dbates@bwh.harvard.edu

Key words: Pandemics, quality, safety,

ACCEPTED MANUSCRIPT

The COVID-19 pandemic created a major health crisis in the North American region, with the U.S. having by far the highest number of deaths, followed by Mexico, with much lower numbers in Canada (Figure 1). This created havoc especially in cities involved early, such as New York City. The first peak occurred in April 2020, with a second, higher peak in mid-January 2021.

The U.S. has, as of March 2021, the highest number of deaths related to COVID worldwide, with approximately 563,000. Canada in comparison has had only 23,000 deaths; the death rate in the U.S. has been 2.8 times that of Canada. Much of this difference can be related to how the U.S. government addressed the pandemic early on, and the failure of leadership to recognize the seriousness of the virus and implement public health measures. In particular, the federal leadership, including the president, did not follow the recommendations of the top public health officials.[1] The president did not take pandemic risk seriously and had eliminated federal support for the group which modeled pandemic-related issues and developed pandemic preparedness plans before it started, leaving the U.S. more vulnerable and slower to respond at the onset of the

© The Author(s) 2021. Published by Oxford University Press in association with International Society for Quality in Health Care.

pandemic.[2] Canada in contrast implemented many public health measures early on and responded more rapidly and effectively, resulting in lower case rates than in the U.S.

Not surprisingly, addressing this crisis resulted in numerous changes in healthcare systems in this region. Perhaps the most obvious was that telehealth received an enormous boost. While most organizations in the U.S. had dragged their feet about adopting telehealth largely because of concerns about reimbursement and provider reluctance to switch to it, once the pandemic started, most outpatient visits were switched to telehealth almost overnight.[3] This put great strains on the supporting services such as the applications handling visits which were not necessarily designed to handle the volume they received. Even in the inpatient setting, infection control needs were such that interactions between patients and staff were dramatically reduced often via telehealth approaches, such as giving patient an electronic tablet and allowing them to interact with a staff member while maintaining distance.

Going forward, this has raised many questions about the proper use of telehealth: what percentage of visits should be virtual? Which types of visits are appropriate for virtual care and which ones are inappropriate? What technology is needed to make this work at scale? How can access to the necessary technologies be made more equitable? Answers to these are still being determined.

From a diagnostic perspective, clinicians were not used to doing evaluations without vital signs or physical examination and this made many of them uncomfortable. There were justifiable concerns that loss of body cues and touch can limit the patient-doctor relationship. It was often more difficult to organize laboratory testing and radiographs as well as vaccination without face-to-face visits. There were also challenges with the technology itself. The COVID healthcare coalition identified that 64% of participants were concerned about technical issues.[4] The user

ACCEPTED MANUSCRIPT

interfaces were often confusing. Sometimes there were issues with connectivity and interactions would drop. Providers often found that they needed to spend substantial time getting patients properly connected at the beginning of an interaction and sometimes that would take as much time as the entire visit. In addition, there were issues with payment. Reimbursement for telehealth varies considerably by insurance provider. The laws around telehealth in United States make visits across state lines difficult and between some states it is considered illegal to practice across state lines Often video visits are paid for but not telephonic telehealth visits, even though it is not clear there is more value associated with the visit video visits. Digital solutions were also implemented to ensure provider safety. Many hospitals implemented the use of digital tools to assess whether employees had COVID symptom on a given day, which turned out to be very helpful for preventing further viral spread.

Changes also occurred within physical hospital infrastructure and flow. Hospitals had to adapt their testing and admission procedures much more rapidly than they had previously been used to. Many doubled the size of their intensive care units and set up designated COVID units to try and decrease the risk of transmission to patients in other departments. Doing this quickly as was needed was very difficult. There were major shortages of personal protective equipment (PPE) and other key supplies and problems with the supply chain were exposed. Many hospitals stopped doing elective procedures to cope with the large volume of COVID patients and, because this is a primary revenue source, this created financial difficulties.

Patients with chronic diseases were getting relatively little attention during the early stages of the pandemic.[5] This resulted in worse outcomes for patients with progressive conditions such as congestive heart failure and coronary ischemia. There were numerous instances in which new care models had to be put in place, for example ventilation of patients in rooms not designed for

ACCEPTED MANUSCRIPT

intensive care, nearly overnight in certain cases. The speed that was needed was very different than what most systems were used to. Governance had to be changed dramatically to enable more rapid decision making and the pandemic made nearly all organizations appreciate the value of having robust data to make important decisions for example about whether to open additional COVID units or whether additional ventilators might be needed.

Overall, the ability to respond to changes did improve dramatically over the course of the pandemic. Not surprisingly, the most difficult part was the first major peak when it was unclear how many people would become ill and whether the system would be overwhelmed. This was especially problematic in places that had very large numbers of cases early like New York City. However, during later stages of the pandemic, when forecasting of cases and capacity assessments were more accurate, most areas managed well.

This pandemic has also proved to be very stressful for clinicians, especially those on the front lines who worked long hours and faced considerable personable risks. Rotenstein et al suggest that organizations should invest in strategies that promote clinician wellbeing, and that this should integrate six principles which resonate for pandemic management more broadly, "1) agility and responsiveness, 2) supportive culture, 3) transparent communication, 4) connection to meaningful work, 5) supportive systems of working, and 6) promotion of equity."[6]

A Boston Consulting Group report published at the end of 2020 suggested that they were 5 key lessons to learn from around early pandemic response: 1) improve patient health outcomes by defining appropriate care for specific population segments, 2) implement new care models realigning resources in order to rethink the way that health care is delivered, 3) apply digital solutions more widely, 4) revamp governance and policy to define shared goals and improve decision-making and 5) adopt a standardized data and analytics system across a broader range of

ACCEPTED MANUSCRIPT

use cases. [7] All of these would be prudent for future pandemics, though some such as redefining governance and adopting more standard data and analytics approaches take time and perseverance.

Going forward, it seems almost certain that changes from COVID will affect the system in profound ways in the future. There will be much more use of telehealth than previously. Many patients found it very efficient, especially as they became much more used to interacting with the healthcare system through tools like patient portals, which were a primary method for accessing the system during the height of the pandemic. Perhaps more importantly, organizations have recognized the need to streamline some of their policies and develop the ability to act more quickly. They also need to pay attention to the needs of their workforce, which are strained by a crisis like this. Finally, it is likely that health care systems will place more emphasis on investing in digital solutions and on having access to good data in analytics that has been the case in the past. Overall, there are many lessons for the system to absorb, mainly about the need to be nimble in such situations, and the vital importance of having good data for decision-making.

ACCEPTED MANUSCRIPT

Figure 1. Case rates for COVID for the U.S. and Canada. Analytics like this increasingly became available during the pandemic and have been very helpful for managing it effectively.

From: Our World in Data[8]

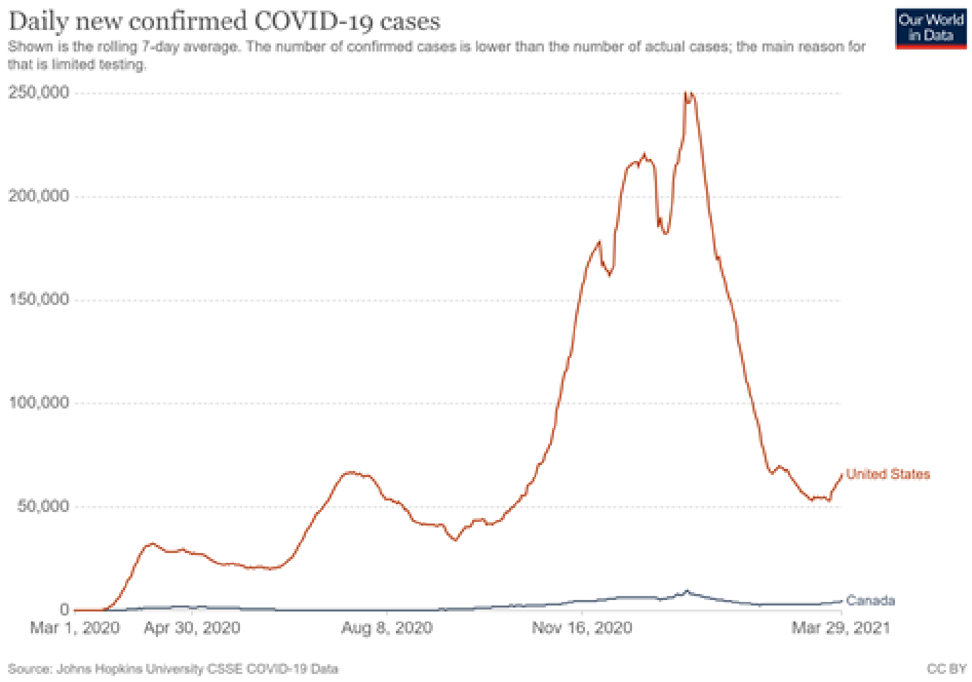

ACCEPTED

References

1. Gupta S, Fauci A, Birx D. Dr. Birx: All deaths over first 100,000 could have been mitigated - CNN Video. CNN. https://www.cnn.com/videos/health/2021/03/27/fauci-birx-covid-war-gupta-intv-sot-vpx.cnn. Published March 27, 2021. Accessed June 2, 2021.

2. Cameron B. I ran the White House pandemic office. Trump closed it. . The Washington Post . https://www.washingtonpost.com/outlook/nsc-pandemic-office-trump-closed/2020/03/13/a70de09c-6491-11ea-acca-80c22bbee96f_story.html. Published March 13, 2020. Accessed June 2, 2021.

3. Fischer SH, Uscher-Pines L, Roth E, Breslau J. The Transition to Telehealth during the First Months of the COVID-19 Pandemic: Evidence from a National Sample of Patients. *J Gen Intern Med*. 2021;36(3):849-851. doi:10.1007/s11606-020-06358-0

4. The Mitre Corporation. COVID-19 Healthcare Coalition. https://c19hcc.org/. Accessed June 2, 2021.

5. Wright A, Salazar A, Mirica M, Volk LA, Schiff GD. The Invisible Epidemic: Neglected Chronic Disease Management During COVID-19. *J Gen Intern Med*. 2020;35(9):2816-2817. doi:10.1007/s11606-020-06025-4

6. Rotenstein LS, Braunfelds D, Sliwka D. Six Principles To Ensure Clinician Well-Being: Lessons From COVID-19's Darkest Days For A New Way Of Working. Health Affairs. https://www.healthaffairs.org/do/10.1377/hblog20210324.54806/full/. Published March 29, 2021. Accessed June 2, 2021.

7. Chandran P, Torelm M, Yong C, Clawson J. Five Lessons from the Pandemic for Health Care Systems. https://www.bcg.com/publications/2020/five-lessons-for-health-care-

ACCEPTED MANUSCRIPT

systems-covid-19. Published December 3, 2020. Accessed June 2, 2021.

8. Our World in Data. Daily new confirmed COVID-19 cases per million people. https://ourworldindata.org/covid-cases. Published March 29, 2021. Accessed June 2, 2021.

ACCEPTED MANUSCRIPT
